# Widespread *Trypanosoma cruzi* infection in government working dogs along the Texas-Mexico border: Discordant serology, parasite genotyping and associated vectors

**DOI:** 10.1371/journal.pntd.0005819

**Published:** 2017-08-07

**Authors:** Alyssa C. Meyers, Marvin Meinders, Sarah A. Hamer

**Affiliations:** 1 Department of Veterinary Integrative Biosciences, Texas A&M University, College Station, Texas, United States of America; 2 Office of Health Affairs, Department of Homeland Security, Washington D.C., United States of America; Universidade Federal de Minas Gerais, BRAZIL

## Abstract

**Background:**

Chagas disease, caused by the vector-borne protozoan *Trypanosoma cruzi*, is increasingly recognized in the southern U.S. Government-owned working dogs along the Texas-Mexico border could be at heightened risk due to prolonged exposure outdoors in habitats with high densities of vectors. We quantified working dog exposure to *T*. *cruzi*, characterized parasite strains, and analyzed associated triatomine vectors along the Texas-Mexico border.

**Methodology/Principle findings:**

In 2015–2016, we sampled government working dogs in five management areas plus a training center in Texas and collected triatomine vectors from canine environments. Canine serum was tested for anti-*T*. *cruzi* antibodies with up to three serological tests including two immunochromatographic assays (Stat-Pak and Trypanosoma Detect) and indirect fluorescent antibody (IFA) test. The buffy coat fraction of blood and vector hindguts were tested for *T*. *cruzi* DNA and parasite discrete typing unit was determined. Overall seroprevalence was 7.4 and 18.9% (n = 528) in a conservative versus inclusive analysis, respectively, based on classifying weakly reactive samples as negative versus positive. Canines in two western management areas had 2.6–2.8 (95% CI: 1.0–6.8 p = 0.02–0.04) times greater odds of seropositivity compared to the training center. Parasite DNA was detected in three dogs (0.6%), including TcI and TcI/TcIV mix. Nine of 20 (45%) *T*. *gerstaeckeri* and *T*. *rubida* were infected with TcI and TcIV; insects analyzed for bloodmeals (n = 11) fed primarily on canine (54.5%).

**Conclusions/Significance:**

Government working dogs have widespread exposure to *T*. *cruzi* across the Texas-Mexico border. Interpretation of sample serostatus was challenged by discordant results across testing platforms and very faint serological bands. In the absence of gold standard methodologies, epidemiological studies will benefit from presenting a range of results based on different tests/interpretation criteria to encompass uncertainty. Working dogs are highly trained in security functions and potential loss of duty from the clinical outcomes of infection could affect the work force and have broad consequences.

## Author summary

Chagas disease, a potentially deadly cardiac disease of humans, canines and other mammals is caused by the parasite *Trypanosoma cruzi*. The parasite is primarily transmitted to dogs by ingestion of infected triatomine ‘kissing bug’ vectors or through contact with the insect’s feces. Previous studies concluded that stray and shelter dogs are at high risk of infection in the southern U.S. We proposed that high-value U.S. government working dogs along the Texas-Mexico border may also be at high risk because of their activities in regions with established, infected vector populations. We sampled 528 working dogs along the Texas-Mexico border, and found that 7.4–18.9% of dogs were positive for *T*. *cruzi* antibodies and a small proportion (0.6%) also had parasite circulating in the blood. We collected two species of kissing bugs from the canine environments and used molecular approaches to determine that 45% were positive for *T*. *cruzi* and the majority had recently fed on canines. We highlight the need for better diagnostic tools for canine Chagas disease research and diagnosis. The widespread burden of *T*. *cruzi* infection in the government working dogs could be associated with far-reaching consequences for both animal and human well-being.

## Introduction

Chagas disease, a potentially deadly cardiac disease of humans and dogs, is caused by the flagellated protozoan parasite *Trypanosoma cruzi*. The parasite is transmitted by infected hematophagous triatomine insects, commonly known as ‘kissing bugs’. Chagas disease is estimated to infect nearly 6 million people throughout Latin America, and occurs across the southern US in enzootic cycles [1,2], where raccoons and other wildlife serve as reservoirs [2,3]. In many areas of Latin America, such as in the Gran Chaco ecosystem, domestic dogs are an important reservoir of *T*. *cruzi* and domestic vectors that fed on dogs showed higher infection prevalence than vectors that fed on other domestic hosts [4,5]. The importance of canines in the *T*. *cruzi* transmission cycle in the US is not yet understood.

The occurrence of *T*. *cruzi* infected canines in the USA is especially high in the state of Texas [1,6,7], where 439 cases were reported across 58 counties between 2013–2015 when there was mandatory reporting of *T*. *cruzi* infected dogs [8]. Texas harbors at least seven established species of triatomine vectors capable of transmitting *T*. *cruzi* [3] and infected wildlife are widespread [1]. The high frequency of canines infected with *T*. *cruzi* likely reflects robust enzootic transmission in the state. Outside of Texas, dogs infected with *T*. *cruzi* have been reported in Louisiana [9,10], Oklahoma [11,12], Tennessee [13] and Virginia [14]. Across the studied populations, apparent seroprevalence ranged from 3.6–57.6% and predispositions of infection status with certain breeds or types of dogs do not appear to be strong, with hunting dogs, working dogs, household pets, shelter and stray dogs all impacted [6,7,9,12,14,15].

*T*. *cruzi* infection can occur by vector-mediated transmission through the introduction of infected bug feces into the bite site or mucous membrane or through the ingestion of infected bugs or their feces [5]. Additionally, congenital transmission may occur [3]. Dogs are more likely to become infected than humans [16,17], which could be from dog’s affinity to consume bugs [12,18–21]. *T*. *cruzi*-infected dogs may be asymptomatic or may develop debilitating acute or chronic cardiac disease, characterized by myocarditis, hepatomegaly, ascites, cardiac dilatation, or sudden death [22]. There are currently no vaccinations or approved anti-parasitic treatments for *T*. *cruzi* infections in dogs in the US, and infected dogs are treated symptomatically.

The Department of Homeland Security (DHS) of the US government manages over 3,000 working dogs in various capacities including the Transportation Security Authority, Coast Guard, Secret Service, Federal Protective Services, Customs and Border Protection, and Federal Operations. These dogs are highly trained in working duties performed in the indoor and outdoor environment including search and rescue functions as well as detection of concealed persons, narcotics, or explosives. DHS working dogs may be at increased risk for contact with vector species from working and sleeping outdoors. Some of the working dogs are kept in group kennels, which have previously been shown to be a risk factor for *T*. *cruzi* infection [7]. Their working environment could further be an attractant to the vector, where there is high vehicle traffic emitting CO_2_- a known attractant [23], bright lights at night, and concentrations of animals and people in otherwise rural areas. In order to provide a baseline for conducting clinical assessments and developing disease management strategies, we conducted a seroepidemiological investigation to quantify the prevalence of *T*. *cruzi* infection in populations of working dogs along the Texas-Mexico border. Additionally, we aimed to determine the infection status and feeding patterns of triatomine vectors in the environments where these dogs work and are kenneled.

## Methods

### Ethics statement

All canine samples were collected in adherence with animal use protocols approved by Texas A&M University’s Institutional Animal Care and Use Committee on 08/17/2015 under the number 2015–0289. Written consent was received for each canine sampled from DHS personnel.

### Study population- DHS working dogs

Sampled DHS working dog breeds were predominantly Belgian Malinois and German Shepherds. Most dogs were bred in Europe, and less commonly dogs came from vendors within Texas or other parts of the US. Dogs receive over 6 months of training at either a training facility in El Paso, Texas, or Front Royal, Virginia, and specialize in various jobs such as track and trail, detection of humans, narcotics, currency, or agricultural products, and search and rescue. After training, dogs are typically assigned to a specific management area and have limited travel. The dogs in our study perform working duties either immediately adjacent to the geopolitical border (ports of entry) or north of the border (checkpoints). Off-duty canines are either kenneled individually at their handler’s residence or in a group kennel. Residential kennels are indoor-outdoor metal kennels raised 2 feet from the ground, giving the dog the option of sleeping inside or outside. Group kennels are indoor-outdoor, concrete kennels, and dogs are confined inside during the night.

### Sample collection

**Fig 1:**
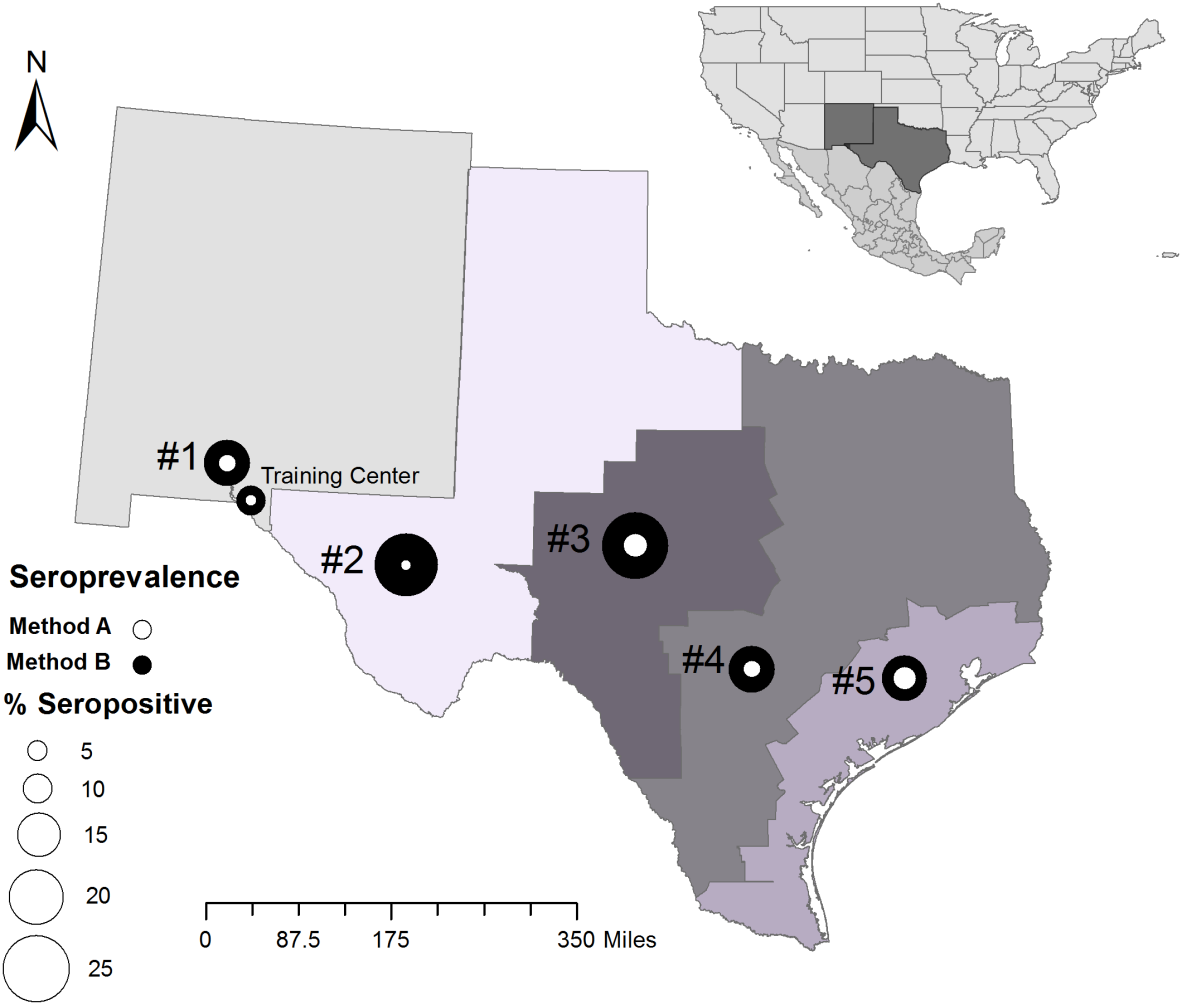
Seroprevalence of government working dogs along the Texas-Mexico border by two methods. Government working dogs were sampled from five management areas (#1–5) and a training center along the Texas-Mexico border in 2015–2016; management area #1 spans New Mexico and Texas. Seroprevalence was calculated using Method A- a conservative analysis in which very faint serological bands were interpreted as negative, and Method B- an inclusive analysis in which very faint serological bands were interpreted as positive. Map was created using ArcGIS and with Texas, Mexico, and New Mexico base layers downloaded from www.arcgis.com and the U.S. Department of Commerce, U.S. Census Bureau, Geography Division, Cartographic Products and Services Branch.

We used a cross sectional study design to collect blood samples from DHS working dogs during November 2015 and April 2016. Working dogs were sampled from all 5 management areas, with a goal of sampling at least 60% of the dogs that occurred within each management area. Additionally, we sampled DHS canines that were in training at a training facility in management area #1 (Fig 1). Sample criteria included dogs over 6 months in age and on active duty or in training. Demographic information was collected on all dogs sampled including age, sex, breed, canine job, sleeping location and station of duty. A minimum of 1 ml of blood was collected by venipuncture and aliquoted into serum and EDTA tubes.

### Serological methods

Samples were screened for anti-*T*. *cruzi* antibodies by Chagas Stat-Pak rapid immunochromatographic test (ChemBio, NY) which was designed for use in humans and has been validated in dogs [9]. Stat-Pak assay uses three *T*. *cruzi* recombinant antigens that are bound to the assay membrane solid phase. Serum or plasma samples were tested according to manufacturer’s protocol and read for result determination after 15 minutes. Tests were considered negative when no color developed and positive when a clear line developed. Additionally, very faint bands that were not perceptible enough to be consider a clear positive, yet with some low level of color development to differentiate them from negative, were tracked as ‘inconclusive’ and subjected to additional testing.

All positive or inconclusive samples as determined by Stat-Pak plus 10% of the negatives were tested by both indirect fluorescent antibody (IFA) test and Trypanosoma Detect (InBios, International, Inc., Seattle, WA). IFA detects anti-*T*. *cruzi* IgG antibodies and was performed by the Texas Veterinary Medical Diagnostic Laboratory (TVMDL, College Station, TX) on serum or plasma samples. Titer values of 20 or higher were considered positive per TVMDL standard protocol; this titer value cutoff has also been used in human medicine [24]. IFA readers were blinded to previous serologic results. Trypanosoma Detect is a rapid immunochromatographic dipstick assay that employs a multi-epitope recombinant antigen for the detection of anti-*T*. *cruzi* antibodies. The Trypanosoma Detect test was designed for use in humans but has been found to have high sensitivity and specificity for use in dogs [25]. Serum or plasma were tested according to manufacturer's protocol and read for result determination after 20 minutes. Test results were scored as positive, inconclusive, or negative using the same criteria as described above for the Chagas Stat-Pak. Serological positive status was assigned to samples that tested positive on at least two independent tests.

### Molecular methods

Amplification of parasite DNA from blood samples by real time PCR was performed on all sampled dogs. DNA was isolated from 250 uL of buffy coat by using E.Z.N.A. Tissue DNA kit (Omega Bio-Tek, Norcross, GA). Negative controls (phosphate buffered saline or water template) were included in the DNA extractions and the PCR. To determine if analysis of clot rather than buffy coat may result in a greater ability to detect parasite DNA, we conducted additional work with a subset of samples as follows. From 12 dog samples, we extracted DNA from 1 mL of clot for PCR analysis. These 12 dogs comprised 10 that were seropositive and PCR negative based on buffy coat; 1 that was seropositive and PCR positive based on buffy coat; and 1 that was seronegative and PCR positive based on buffy coat analysis.

Samples were first screened for presence of *T*. *cruzi* satellite DNA using the Cruzi 1/2 primer set and Cruzi 3 probe in a real-time assay to amplify a 166-bp segment of a repetitive nuclear DNA [26, 27]. Reactions consisted of five microliters of extracted DNA, primers I and II each at a concentration of 0.75 μM, 0.25 μM of probe, and iTaq University Probes Supermix (BioRad Laboratories, Hercules, CA), in a 20 μL reaction volume. Previously published thermocycling parameters were followed except with a 3-minute initial denaturation using a Stratagene MxPro3000 (Agilent Technologies, Santa Clara, CA). *T*. *cruzi* DNA extracted from isolate Sylvio X10 CL4 (ATCC 50800, American Type Culture Collection [ATCC]) was used for a positive control. Machine-calculated thresholds and reaction curves were visually checked for quality. Samples with Ct values less than 34 were considered suspect positive and subjected to further testing.

Suspect positive samples by qPCR were run on a second, independent PCR using *T*. *cruzi* 121/122 primers to amplify a 330-bp region of kinetoplast DNA [28,29]. Reactions included 1μL template DNA, primers at final concentrations of 0.75 μM each, and FailSafe PCR Enzyme Mix with PreMix E (Epicentre, Madison, WI) in a final reaction volume of 15 μL. Amplicons were visualized on 1.5% agarose gels stained with GreenGlo safe DNA dye (Denville Scientific Inc., Metuchen, NJ). Samples that yielded a band of the appropriate size were interpreted as positive in this assay. Parasite positive dogs were defined as those that tested positive on both the rt-PCR screening and the secondary PCR assays.

### Determination of *T*. *cruzi* strain types

We used a multiplex quantitative, real time PCR to determine *T*. *cruzi* discrete taxonomic unit (DTU) of samples that were positive or suspect positive on the screening assay based on amplification of the nuclear spliced leader intergenic region (SL-IR) [30]. Using a QIAGEN Multiplex PCR Kit (QIAGEN, USA) reactions were performed using 2μL template DNA in a final volume of 20 μl and run on a BioRad CFX96 (Hercules, CA, USA). The only deviation from the previously described protocol was the extension of cycles from 40 to 45 and substitution of dyes as previously described [7]. Positive controls consisted of DNA from triatomines collected across Texas that were previously characterized as infected with TcI or TcIV based on amplification and sequencing of the TcSC5D gene [31]. Samples with Ct values less than 34 were considered positive, and fluorescence signal determined the strain type.

### Vector collection and testing for *T*. *cruzi* and bloodmeal analysis

Triatomine bugs were opportunistically collected by dog handlers in summer 2016 from group kennels, outside handler’s residence around canine housing, and at stations where dogs worked. To encourage collections, outreach materials with photos of triatomines and look-alike species were disseminated by email and in printed format to dog handlers prior to the summer peak of adult triatomine activity. Bugs were identified to species using morphologic features [32] and sexed. After bugs were washed in 10% bleach solution and rinsed in distilled water, sterile instruments were used to dissect the bugs, isolate hindgut material and evidence of a recent bloodmeal was noted. DNA was extracted from hindguts and tested for *T*. *cruzi* DNA and determination of *T*. *cruzi* DTU using the same methods as the above testing of dog samples. In order to determine the source of recent bloodmeals, hindgut DNA was subjected to PCR amplification of vertebrate cytochrome B sequences using previously published primers and cycling conditions [33,34]. Reactions included 3 μL template DNA, primers at final concentrations of 0.66 μM each, and FailSafe PCR Enzyme Mix with PreMix E (Epicentre, Madison, WI) in a final reaction volume of 50 μL. Amplicons were visualized on 1.5% agarose gel, prepared for sequencing using ExoSAP-IT (Affymetrix, Santa Clara, CA, USA), and Sanger sequencing was performed (Eton Bioscience Inc., San Diego, CA, USA). Resulting sequences were compared to existing sequences using Basic Local Alignment Search Tool (National Center for Biotechnology Information, US National Library of Medicine). In recognition of the potential for contamination from the environment, samples that aligned to human were re-run on another PCR assay to provide a secondary line of evidence.

### Statistical methods

Due to the uncertainty of sample serostatus associated with the inconclusive band development, antibody-positive dogs were defined using two methods; a) in the conservative method, inconclusive band development was interpreted as negative, and b) in the inclusive method, inconclusive band development was interpreted as positive. In the absence of gold standard serological methodology, these two different criteria of positivity (method A and B) were analyzed separately to provide a range of results.

To evaluate the relationship between potential risk factors and the serostatus of canines, data were imported into R software [35] for analysis. Assessed variables were dog age (young = 6 months to <3 years, middle age = ≥ 3 years to <6 years, senior = ≥ 6 years), sex, breed, sleeping location (individual kennel at handler’s residence or group kennel) and management area (locations 1–5 or training center). Due to the small sample size of dogs in some jobs, canine job was dichotomized based on type of detection. Bivariable analysis using the chi-squared or Fisher’s exact was used to identify putative risk factors. Factors with a p≤ 0.25 from the initial screening were used in a logistic regression model, while controlling for management area as a random effect. Generalized linear mixed models were calculated and factors with values of p < 0.05 were considered significant. Odds ratios and 95% confidence intervals were calculated. To determine variation in seroprevalence across management areas, a logistic regression model was used in which the training center served as the referent to which all five management areas were compared. Kappa index was used to test the agreement between each pairwise combination of the results of the three serological assays for the samples that were tested on all three assays; this sample set was biased toward Stat-Pak positive samples.

## Results

A total of 528 dogs from along the Texas-Mexico border were evaluated using a variety of serologic and molecular techniques to detect *T*. *cruzi* exposure and infection. Distribution of samples among the five management areas ranged from 47 (8.9%) to 135 (25.6%), and 86 (16.3%) dogs were sampled from the training center. The most common breeds were Belgian Malinois and German Shepherd, which together comprised 86% of the sampled dogs, with Dutch Shepherds, Sable Shepherds, Groenendael and Labrador Retriever comprising the remainder. Age ranged from 6 months to 13 years with a median of 4.47 and a mean of 4.79. There were 351 males (66.5%) and 177 females (33.5%). Of the dogs sampled, 55.9% spend their off-duty time in individual residential kennels whereas 44.1% were group kenneled. The sample sizes of dogs within each canine job category or management unit are not disclosed because it is law enforcement sensitive information.

### Serological results interpreted using method A: Conservative method

In considering inconclusive bands on immunochromatographic tests as negative, 39 of 528 (7.4%) of dogs were seropositive for antibodies to *T*. *cruzi* on at least 2 assays. Across management areas and the training center, seroprevalence ranged from 4.3% to 10% (Fig 1). In the bivariable analysis, *T*. *cruzi* seroprevalence was significantly different across dog breed (p = 0.03), with seroprevalence of German Shepherds being lowest (3.7%) and ‘other’ breeds being highest (14.3%; Table 1). Dogs that spent off-duty time in residential kennels had a significantly higher seroprevalence (29/295, 9.8%, p = 0.02) than those that were group-kenneled (10/233, 4.3%). Seroprevalence was significantly different among age groups (p = 0.04), where senior dogs had a seroprevalence of 10.4%, middle age dogs a seroprevalence of 7.9% and young dogs 3.2%. Seroprevalence did not vary significantly by sex or canine job.

**Table 1:** Results of bivariable analysis of potential risk factors for seropositive government working dogs (defined by method A and B) in five management areas and training center along the Texas-Mexico border, 2015–2016.

		Method A: Conservative Method			Method B: Inclusive Method		
Variable	Sample size No. (%)	Seropositive No. (%)	Seronegative No. (%)	P-value	Seropositive No. (%)	Seronegative No. (%)	P-value
**Sex**				0.39			0.6
Female	177 (33.5)	16 (9.0)	161 (91.0)		37 (20.9)	140 (79.1)	
Male	351 (66.5)	23 (6.5)	328 (93.4)		64 (18.2)	287 (81.8)	
**Breed**				0.03			0.86
Belgian Malinois	267 (50.6)	23 (8.6)	244 (91.4)		54 (20.2)	213 (79.8)	
German Shepherd	188 (35.6)	7 (3.7)	181 (96.3)		32 (17.0)	156 (83.0)	
Dutch Shepherd	31 (5.9)	3 (9.7)	28 (90.3)		6 (19.4)	25 (80.6)	
Other	42 (8.0)	6 (14.3)	36 (85.7)		9 (21.4)	33 (78.6)	
**Canine Job**^a^				0.19			0.18
Detection A		(8.3)	(91.7)		(20.5)	(79.5)	
Detection B		(4.2)	(95.8)		(13.7)	(86.3)	
**Sleeping location**				0.02			0.09
Residential	295 (55.9)	29 (9.8)	266 (90.2)		65 (22.0)	230 (78.0)	
Group Kennel	233 (44.1)	10 (4.3)	223 (95.7)		36 (15.4)	197 (84.6)	
**Age**				0.04			0.18
Young	156 (29.5)	5 (3.2)	151 (96.8)		22 (14.1)	134 (85.9)	
Middle Age	190 (36.0)	15 (7.9)	175 (92.1)		39 (20.5)	151 (79.5)	
Senior	182 (34.5)	19 (10.4)	163 (89.6)		39 (21.4)	143 (78.6)	

^a^ Sample sizes of dogs in each canine job as well as the specific detection abilities of dogs are not disclosed because this information is law enforcement sensitive.

Multivariable logistic regression analysis showed a significant association (odds ratio [OR] 0.41, 95% CI 0.17–0.99, p = 0.047, Table 2) between breed and seropositive dogs, after controlling for management areas as a random effect (Table 2), in which German Shepherds were associated with a significantly lower seroprevalence (3.7%) than Belgian Malinois (8.6%). No significant association was found between age, job, or sleeping location and seroprevalence.

**Table 2:** Association between seropositive dogs (defined by method A and B) and age, breed, job and sleeping location.

	Method A: Conservative Method			Method B: Inclusive Method		
Variable	Odds Ratio	95% CI	P-value	Odds Ratio	95% CI	P-value
**Age**						
Young	referent			referent		
Middle Age	2.03	0.69–5.96	0.19	1.4	0.77–2.56	0.27
Senior	2.34	0.78–7.02	0.13	1.37	0.72–2.58	0.33
**Sleeping location**						
Residential	referent			referent		
Group kennel	0.54	0.24–1.24	0.15	0.76	0.46–1.25	0.28
**Canine Job**						
Detection A	referent			referent		
Detection B	0.77	0.27–2.19	0.62	0.85	0.45–1.60	0.61
**Breed**						
Belgian Malinois	referent			*Not included in model*		
German Shepherd	0.41	0.17–0.99	**0.04**			
Dutch Shepherd	1.19	0.33–4.32	0.78			
Other Breed	1.71	0.62–4.69	0.29			

### Serological results interpreted using method B: Inclusive method

In considering inconclusive bands on serologic tests as positive, 100 of 528 (18.9%) of dogs were seropositive for antibodies to *T*. *cruzi* on at least 2 assays. Seroprevalence ranged from 11.6% to 26.7% across management areas and the training center (Fig 1). When running bivariable analysis, dogs that spent off-duty time in residential kennels (65/295, 22%) were marginally (p = 0.09, Table 2) more likely to be seropositive than dogs sleeping at a group kennel (36/233, 15.4%). Seroprevalence did not vary significantly by age, breed, sex or canine job.

Multivariable logistic regression analysis showed that there was no association between age, job, or sleeping location and seroprevalence. Backwards elimination was performed and when only age was included in the model there was a marginal association in which old dogs had a higher seroprevalence (39/182, 21.4%) than young dogs (22/156, 14.1%; p = 0.09), after controlling for management areas as a random effect.

### Seroprevalence across management areas

While seroprevalence did not significantly differ across management areas and the training center when positivity was defined according to Method A, dogs from management area #2 (OR 2.6, 95% CI 1.0–6.7, p = 0.04) and #3 (OR 2.8, 95% CI 1.2–6.8, p = 0.02) had significantly higher seroprevalence compared to the training center when seropositivity was determined according to Method B (Table 3). This indicates that area #2 and #3 were both associated with many samples that produced very faint (inconclusive) bands on the immunochromatographic tests.

**Table 3:** Association between management areas or training center and seropositive dogs with seropositivity defined using method A (including very faint serological bands as negative) and method B (including very faint serological bands as positive).

	Method A: Conservative Method				Method B: Inclusive Method			
Management Area	Seropositive No. (%)	Odds Ratio	95% CI	P-value	Seropositive No. (%)	Odds Ratio	95% CI	P-value
# 1	10 (7.4)	1.6	0.5–6.1	0.42	25 (18.5)	1.7	0.8–4.0	0.17
# 2	2 (4.3)	0.9	0.1–4.9	0.92	12 (25.5)	2.6	1.0–6.7	**0.04**
# 3	6 (10.0)	2.3	0.6–9.3	0.22	16 (26.7)	2.8	1.2–6.8	**0.02**
# 4	7 (7.3)	1.6	0.5–6.3	0.46	18 (18.8)	1.8	0.8–4.2	0.18
# 5	10 (9.6)	2.2	0.7–8.2	0.20	19 (18.3)	1.7	0.8–4.0	0.20
Training Center	4 (4.7)	referent			10 (11.6)	referent		

### Discordant serology

In comparing the results across all three serological testing platforms (Table 4), all IFA positive samples are positive on Trypanosoma Detect, and all but two samples are Stat-Pak positive-both of these samples having a titer of 20. When comparing the IFA negative samples 71.3% are positive or inconclusive on Stat-Pak and 48.4% are positive or inconclusive on Trypanosoma Detect. From the 528 dog samples in the study, 215 samples were tested on all three serology assays. Overall test agreement ranged from slight to moderate agreement based on the Kappa Indices (Table 5), with agreement between tests being better when interpreting immunochromatographic test results using the conservative method A (kappa range 0.37–0.48) compared to inclusive method B (kappa range 0.05–027). The best agreement was using method A between Stat- Pak and Trypanosoma Detect, with a Kappa index of 0.48 (moderate agreement).

**Table 4:** Comparison of results from three serological assays for the detection of anti-*T*. *cruzi* antibodies: Chagas Stat-Pak rapid immunochromatographic test (ChemBio, NY), indirect fluorescent antibody (IFA) test performed by the Texas Veterinary Medical Diagnostic Laboratory (TVMDL, College Station, TX) and Trypanosoma Detect (InBios, International, Inc., Seattle, WA). Number of samples with positive, inconclusive, or negative results on each test are given over the total number of samples with specified IFA endpoint titers.

IFA Titer^a^	Stat-Pak			Trypanosoma Detect		
	Pos.	Inconclusive	Neg.	Pos.	Inconclusive	Neg.
0	34/196	106/196	56/196	46/196	49/196	101/196
20	1/3	1/3	1/3	3/3	0	0
160	3/3	0	0	3/3	0	0
320	9/9	0	0	9/9	0	0
640	4/4	0	0	4/4	0	0

^**a**^no samples had a titer of 40 or 80

**Table 5:** Kappa index comparing three serological assays; Chagas Stat-Pak rapid immunochromatographic test (ChemBio, NY), indirect fluorescent antibody (IFA) test and Trypanosoma Detect (InBios, International, Inc., Seattle, WA). Panels A-C represent Kappa Index when interpreting immunochromatographic test results using the conservative method A (including very faint serological bands as negative); Panels D-F represent Kappa Index when interpreting immunochromatographic test results using the inclusive method B (including very faint serological bands as positive).

**A**.	IFA +	IFA -	Total
Stat-Pak +	17	34	51
Stat-Pak -	2	162	164
Total	19	196	215
Moderate agreement:	**0.41**		
**B**.	IFA +	IFA -	Total
Trypanosoma Detect +	19	46	65
Trypanosoma Detect -	0	150	150
Total	19	196	215
Fair agreement:	**0.37**		
**C**.	Stat-Pak +	Stat-Pak -	Total
Trypanosoma Detect +	36	29	65
Trypanosoma Detect -	15	135	150
Total	51	164	215
Moderate agreement:	**0.48**		
**D**.	IFA +	IFA -	Total
Stat-Pak +	18	140	158
Stat-Pak -	1	56	57
Total	19	196	215
Slight agreement:	**0.05**		
**E**.	IFA +	IFA -	Total
Trypanosoma Detect +	19	95	114
Trypanosoma Detect -	0	101	101
Total	19	196	215
Slight agreement:	**0.16**		
**F**.	Stat-Pak +	Stat-Pak -	Total
Trypanosoma Detect +	98	16	114
Trypanosoma Detect -	60	41	101
Total	158	57	215
Fair agreement:	**0.27**		

Of the 57 randomly-selected Stat-Pak negative samples that were subjected to additional serologic testing, one was positive on both IFA (titer 20) and Trypanosoma Detect; this sample was counted as positive in the seroprevalence estimates. Nine (15.8%) samples that were both Stat-Pak and IFA negative were positive on Trypanosoma Detect; these dogs were counted as negative in the seroprevalence estimates, but could be false negatives. When applying this prevalence of potential false negatives to the total number of dogs that were negative by Stat-Pak, an additional 49 dogs are extrapolated to be potential false negatives; including these samples as positive would increase seroprevalence to 15.9% (84 dogs total) by conservative method A, and 25.4% (149 dogs total) by inclusive method B.

Inconclusive bands were reported from 108 (20.5%) samples screened on Chagas Stat-Pak. When tested on IFA only 1 (0.9%) inconclusive tested positive with a titer of 20. When inconclusive samples were run on Trypanosoma Detect, 37 (29.6%) had inconclusive bands on Trypanosoma Detect, 20 (18.5%) were positive, and 51 (47.2%) were negative.

### Molecular detection of parasite DNA and *T*. *cruzi* strain types

*T*. *cruzi* DNA was detected in the buffy coat fraction of the blood in three of 528 (0.6%) dog samples according to our diagnostic method which included amplification in both a screening and confirmatory assay. The first PCR-positive dog was sampled from area # 5 in November and was positive for antibodies by all three serology assays with a relatively high titer (640) on IFA. Using the multiplex real time PCR to determine *T*. *cruzi* DTUs, we found that this dog harbored DTU TcIV. The second PCR-positive dog was from the canine training center, sampled in April, positive on all serology assays with a titer of 320 and harbored a mix TcI/TcIV. The third dog was from area # 2, sampled in April, was negative by all serological assays, and strain type could not be determined. When this PCR positive yet serologically-negative dog was included in binomial analysis of risk factors and the logistical regression model, no difference was found in significant associations. The subset of 12 samples that were subjected to an additional DNA extraction from 1mL of clot produced PCR results that were identical to the results obtained from the 250 uL buffy coat extractions with the exception of the sample from the seronegative, buffy coat-positive sample. This sample was negative based on clot analysis.

### Infection of triatomine vectors with *T*. *cruzi*

In the summer of 2016, a total of 20 adult triatomine bugs of two species (18 *Triatoma gerstaeckeri* and 2 *T*. *rubida)* were opportunistically collected by canine handlers from three management areas (Table 6). Kissing bugs were collected from stations where dogs and handlers work (n = 6), handler’s residence near canine housing (n = 7), group kennels (n = 4), from the field (n = 2) and 1 bug was removed from a dog while working. Nine (45%) triatomines were positive for *T*. *cruzi* including half of the *T*. *gerstaeckeri* specimens but neither of the two *T*. *rubida* specimens. Of the 9 positive bugs, parasite strain typing revealed DTU TcI in 6, TcIV in 1, and a mixed TcI/TcIV coinfection in 2. From dissection, 13 of the 20 bugs had evidence of a recent blood meal in their hind gut, and 11 of these yielded results after the blood meal analysis protocols, revealing human, canine, coastal-plain toad (*Bufo nebulifer)* and rat (*Rattus rattus)* DNA (Table 6).

**Table 6:** *Triatoma* spp. collected from locations where canines sleep or work were tested for *Trypanosoma cruzi* presence, strain type of *T*. *cruzi* and bloodmeal source were determined.

Triatomine species	Management area	Location	Sex	Bloodmeal source	Strain Type
*T*. *gerstaeckeri*	#3	Group kennel	M	n/a	Negative
*T*. *gerstaeckeri*	#3	Station	F	Dog (*Canis lupus familiaris*)	Negative
*T*. *gerstaeckeri*	#3	Station	F	Dog (*Canis lupus familiaris*)	TcI
*T*. *gerstaeckeri*	#3	Station	M	Human (*Homo sapiens)*	TcI
*T*. *rubida*	#2	Station	F	n/a	Negative
*T*. *gerstaeckeri*	#3	Station	M	n/a	TcI
*T*. *gerstaeckeri*	#3	Group kennel	F	n/a	Negative
*T*. *gerstaeckeri*	#3	Group kennel	M	n/a	TcI
*T*. *gerstaeckeri*	#3	On canine	F	n/a	TcIV
*T*. *gerstaeckeri*	#5	Residential kennel	F	Dog (*Canis lupus familiaris*)	Negative
*T*. *gerstaeckeri*	#5	Residential kennel	M	Dog (*Canis lupus familiaris*)	TcI
*T*. *gerstaeckeri*	#5	Field	F	n/a	Negative
*T*. *gerstaeckeri*	#5	Residential kennel	M	Coastal-Plain toads (*Bufo nebulifer*)	TcI
*T*. *gerstaeckeri*	#5	Field	M	Rat (*Rattus rattus*)	Negative
*T*. *gerstaeckeri*	#5	Residential kennel	F	n/a	TcI/TcIV
*T*. *gerstaeckeri*	#5	Residential kennel	M	Coastal-Plain toads (*Bufo nebulifer*)	Negative
*T*. *gerstaeckeri*	#5	Residential kennel	F	n/a	Negative
*T*. *rubida*	#2	Residential kennel	F	Dog (*Canis lupus familiaris*)	Negative
*T*. *gerstaeckeri*	#3	Station	F	Dog (*Canis lupus familiaris*)	TcI/TcIV
*T*. *gerstaeckeri*	#3	Group kennel	M	Human (*Homo sapiens)*	Negative

## Discussion

We found widespread *T*. *cruzi* infection in government working dogs along the Texas-Mexico border. DHS working dogs play an important role in detection and security functions in the Unites States and the clinical manifestation of infection may be associated with significant future economic and security consequences. We are aware of only two prior epidemiological investigations of *T*. *cruzi* infection in working dogs in the US. In 2007, a serological survey was conducted on military working dogs (MWD) in San Antonio, TX, after veterinarians noted an increase in Chagas disease diagnoses, revealing 8% of the kenneled dogs were positive by IFA [36]. Such findings are of utmost importance in these dogs; in 2009, MWDs deployed in Iraq were evacuated due to cardiac symptoms and diagnosed with *T*. *cruzi* infection leaving troops vulnerable without explosive detection dogs [36]. Recently, populations of working hound dogs in south central Texas that are used for scent detection and track/trail were characterized with an extremely high seroprevalence of 57.6% (n = 85) in which positive dogs were reactive on both Stat-Pak and IFA [7]. The study population also included many dogs with parasite DNA in the blood and other organs, and infected triatomines collected from the dog kennels were determined to have fed on dogs, allowing the authors to conclude that multi-dog kennels can be high risk environments of *T*. *cruzi* transmission [7].

Exposed dogs were present in all five management areas and the canine training school, with an overall apparent seroprevalence of 7.4–18.9%. This seroprevalence is similar to that reported from dogs in Chagas-endemic areas in Latin America including populations in Peru (12.3%) [37], Argentina (45.6%) [38], Panama (11.1%) [39], Costa Rica (27.7%) [19], Yucatan State, Mexico (9.8%-14.4%) [40] and Mexico State, Mexico (10%-15.8%) [41]. Previous epidemiological investigations of *T*. *cruzi* in canines in the US are limited, and most have focused on stray dogs or those sampled from animal shelters, which may be considered as high risk populations due to outdoor activity. A serosurvey of high risk kenneled dogs in southern Louisiana found that 22.1% [9] of dogs tested positive for *T*. *cruzi* antibodies using the same three serology assays performed in this study. A study in Oklahoma sampling shelter dogs and pet dogs concluded that 3.6% dogs were seropositive when testing by radioimmunoprecipitation assay (RIPA) [12]. Earlier studies in southern Texas stray dogs 375 dogs were tested and 7.5% were positive by indirect immunofluorescence [15]. Similarly, across Texas shelter dogs had a seroprevalence of 8.8% when testing dogs on Chagas Stat-Pak [6]. These studies and ours suggest that despite the regular veterinary care, quality food and shelter, highly-valued working dogs can have similar or greater *T*. *cruzi* infection than stray and shelter dogs in the US and free roaming or pet dogs in endemic countries.

Both population-level and individual-level *T*. *cruzi* studies of naturally-infected hosts suffer from a lack of gold standard tests or diagnostic recommendations. Discordance among tests results is prevalent in human and veterinary Chagas diagnostics. For example, a study looking at seroprevalence in people from Veracruz, Mexico used 5 assays and found that test agreements ranged from 0.038–0.798 on the Kappa index [42]. Similarly, using the Kappa index we found a high discordance among serology assays used, with agreement ranging from slight to moderate depending on the interpretation method. Assay discordance could be affected by the single freeze-thaw cycle, or the age of the sample. These diagnostic challenges make it difficult to directly compare seroprevalence across populations and diagnostic methods, and presents a challenge in clinical settings for diagnosis. Two of the three serological tests we used are only available for research use for dogs in the US, and a limited number of commercial laboratories offer canine *T*. *cruzi* diagnostic test services. As in most diagnostic tests, there is some subjectivity in the interpretation of results, and the development of very faint ‘equivocal’ serological bands on both Chagas Stat-Pak and Trypanosoma Detect posed particular complexities in our analysis. The Stat-Pak and Trypanosoma Detect instructions state that band intensity will vary, but faint bands should be interpreted as positive [43,44] and that variation is dependent on the concentration of antibodies present [44]. However, some previous canine studies have counted faint bands as negative [6,9] while others have interpreted them as positive for analysis [45]. Our presentation of a seroprevalence calculated both conservatively (very faint bands interpreted as negative) and inclusively (very faint bands interpreted as positive) is an effort to account for imperfect diagnostics. Until refined *T*. *cruzi* diagnostic tools are available, we encourage transparency in presenting results on single vs. multiple tests across all strengths of test response.

The discordance between test results and the within assay variation could be caused by parasite heterogeneity [45]. *T*. *cruzi* is notably heterogeneous with seven major genotypes or discrete typing units (DTUs) described as TcI-TcVI and TcBat which vary be region [46,47]. Additionally, a notable intra-DTU variability has been found [47,48]. Previous research has found that assay reactivity varies by geographic origin of the patient [49]. O’Connor and others found that strain TcI clusters geographic between North and South America [50]. The Chagas Stat-Pak was validated with human sera from Central America to detect strains circulation in that region [51] and may not be optimized for *T*. *cruzi* clones from Texas. When very faint bands were interpreted as positive (method B), seroprevalence was significantly higher in two western management areas (OR 2.6–2.8, 95% CI: 1.0–6.8 p = 0.02–0.04) compared to the training center (Table 3), whereas this difference was not evident when very faint bands were interpreted as negative (method A). The disproportionate abundance of very faint bands in this geographic area may be driven by differences in the locally-circulating *T*. *cruzi* clones. Diosque et al. performed a genetic survey of *T*. *cruzi* isolates within a restricted geographical area (~300 km^2^) and found five different clones circulating [52]; such findings are clinically and diagnostically relevant because parasite heterogeneity has been shown to cause varying infectivity and immune response [52–54]. In addition, host biological factors (exposure history, coinfection, genetic makeup) could also cause reaction variability within and across serology assays.

Sleeping location (group housed indoors vs. individually housed outdoors) appeared to be independently associated with *T*. *cruzi* status with a higher seroprevalence in dogs sleeping outdoors than indoors by method A (p = 0.02), and marginally significant by method B (p = 0.09) in bivariable analysis. Previous studies have indicated dogs housed outdoors where vector contact is more likely to be at a higher risk for exposure [6,9,39]. Dogs in Tennessee spending 100% of their time outdoors were significantly more likely to be seropositive for *T*. *cruzi* than dogs spend ≤50% of their time outdoors [13]. Seroprevalence did increase with age in both method A and B, but was only significant in bivariable analysis in method A, where senior (>6 years) and middle age (≥ 3 years to <6 years), were more likely to be seropositive than young dogs (<3 years old) (Table 1). This is anticipated in infectious disease since exposure increases with age and has been found in previous studies [7,13,17,55]. We found that German Shepherds were associated with a significantly lower seroprevalence (3.7%) than Belgian Malinois (8.6%) in our study; although the driving factors for this difference are currently unknown, it may relate to host behavior, differences in host immune response, or a physical characteristic.

We found three dogs (0.6%) harbored parasite PCR in their blood, suggesting that these dogs are parasitemic. While two of the three PCR-positive dogs also harbored detectable anti *T-cruzi* antibodies, one did not, suggesting this dog may have been in the acute stage of infection [56]. The two dogs with successfully typed infections harbored DTUs TcIV and a TcI/TcIV mix, consistent with previous studies on dogs in the US [57,58]. Both strain types infect a variety of hosts and vectors in the southern US [3]. DTU TcI is an ancient strain found throughout South and Central America and the predominant strain infecting humans in the US [3], where it is also associated with wildlife reservoirs including opossums (*Didelphis virginiana*) [57]. TcIV is also associated with wildlife, especially raccoons (*Procyon lotor*) [3] and to our knowledge has not been implicated in the small number of typed human infections in the US. This study found a lower prevalence of dogs PCR positive then previous studies, which likely reflects the time of sampling (November and April) when the vector is less active and dogs in Texas are less likely to come in contact with the kissing bug [58]. In recognition of other datasets that have shown that analysis of clot, rather than buffy coat, may afford a greater the chance of detecting parasite DNA [59], we subjected 12 clot samples to PCR and compared results to previous results from analysis of buffy coat. We found that buffy coat and clot results were identical across this subset with the exception of a sample from a single seronegative dog which was positive from buffy coat and negative from clot. Based on this small comparison trial, we suggest that the low frequency of encountering PCR-positive dogs in our study was not due to the blood fraction used in the analysis.

We found an infection prevalence of 45% in the kissing bugs collected from areas where the working dogs frequent, including kennels, stations and handler’s residence, including DTUs TcI, TcIV, and TcI/IV mix. This infection is slightly lower than previous estimates across the state of Texas of 63% and 51% [59,60]. Bloodmeal analysis revealed canine, human, and wildlife DNA within the hindguts of these insects, underscoring the generalist feeding strategies of triatomines that often use the most locally abundant hosts. Strict protocols were used to reduce the risk of contamination of samples by exogenous DNA (i.e., human DNA), including surface sterilization of vectors and dissection of the hindguts. It is biologically plausible that the insects associated with suspected human blood feeding encountered humans at their residence or station or work. A study in California and Arizona that collected bugs by light traps found that 5 of 13 bugs (38%) bugs were positive for a human blood meal, 4 fed on canine and 1 each for rat, pig, chicken and mouse [61]. In Texas, Gorchakov et al. found 65% (n = 62) of bugs positive for human bloodmeal and 32% for canid bloodmeal [62]; in contrast, Kjos et al. found only 1% of vectors (n = 96) collected from residential settings had fed on a human, and 20% on dogs [63]. Larger sample sizes of engorged vectors from the working dog environments will assist in learning the local vector-host interactions that sculpt disease risk.

Using dogs as sentinels has been suggested for targeted vector control programs endemic areas such as Peru [37] and to monitor transmission in Argentina [55]. However, the relative importance of dogs as reservoirs, and whether or not they can be a sentinel species for human disease risk in the US, is unknown. Further, because the triatomines in the US tend not to be colonized within homes, dogs are less likely to be useful sentinels at the household level. Nonetheless, given these infected working dogs signal the presence of infected vectors in the environment, there are public health implications of these findings especially with respect to the human handlers who are exposed to the same environments.

Because not all *T*. *cruzi*-infected dogs will develop disease [21], the prognosis and clinical implications of the widespread presence of *T*. *cruzi*-infected government working dogs along the US-Mexico border is unknown. Nonetheless, the potential loss of duty days resulting in an inadequate canine workforce must be considered. Additionally, given that the canine training school in west Texas (Fig 1) occurs in an area where triatomines are endemic, vector and canine surveillance must be conducted to determine if young dogs may be exposed to the parasite while in training, which would not only have implications for the health of the dog but also potentially afford dispersal of the parasite to the new areas across the US where these dogs are stationed. Understanding the epidemiology of *T*. *cruzi* infection is the first step toward implementing control measures to protect the health of these high-value working dogs.

## Supporting information

S1 Checklist Strengthening the Reporting of Observational Studies in Epidemiology (STROBE) checklist of items that should be included in reports of observational studies. (DOC)
