# Supplementary material for: Widespread Trypanosoma cruzi infection in government working dogs along the Texas-Mexico border: Discordant serology, parasite genotyping and associated vectors
Source: PLoS Negl Trop Dis. 2017 Aug 7;11(8):e0005819. doi: 10.1371/journal.pntd.0005819 (PMC5560752; doi:10.1371/journal.pntd.0005819)
Supplement: S1 Checklist — (DOC) [file pntd.0005819.s001.doc]

STROBE Statement—Checklist of items that should be included in reports of ***cross-sectional studies***

|  | Item No | Recommendation | Page No.** | Relevant text from manuscript |
| --- | --- | --- | --- | --- |
| **Title and abstract** | 1 | (*a*) Indicate the study’s design with a commonly used term in the title or the abstract | 1-3 | “In 2015-2016, we used a cross-section study design to collect blood samples from Department of Homeland Security working dogs in five management areas plus a training center and collected triatomine vectors from canine environments. .” |
|  |  | (*b*) Provide in the abstract an informative and balanced summary of what was done and what was found | 1-3 |  |
| Introduction | | |  |  |
| Background/rationale | 2 | Explain the scientific background and rationale for the investigation being reported | 6 | “DHS working dogs may be at increased risk for contact with vector species from working and sleeping outdoors. Some of the working dogs are kept in group kennels, which have previously been shown to be a risk factor for *T. cruzi* infection [7]. Their working environment could further be an attractant to the vector, where there is high vehicle traffic admitting CO_2_- a known attractant [23], bright lights at night and concentrations of animals and people in otherwise rural areas.” |
| Objectives | 3 | State specific objectives, including any prespecified hypotheses | 6 | “…to provide a baseline for conducting clinical assessments and developing disease management strategies, we conducted a seroepidemiological investigation to quantify the prevalence of *T. cruzi* infection in populations of working dogs along the Texas-Mexico border. Additionally, we aimed to determine the abundance, infection status, and feeding patterns of triatomine vectors in the environments where these dogs work and are kenneled.” |
| Methods | | |  |  |
| Study design | 4 | Present key elements of study design early in the paper | 7-8 | “We used a cross sectional study design to collect blood samples from DHS working dogs during November 2015 or April 2016.Working dogs were sampled from all 5 management areas, with a goal of sampling at least 60% of the dogs that occurred within each management area…” |
| Setting | 5 | Describe the setting, locations, and relevant dates, including periods of recruitment, exposure, follow-up, and data collection | 7,11 | “The dogs in our study perform working duties either immediately adjacent to the geopolitical border (ports of entry) or north of the border (checkpoints). Off-duty canines are either kenneled individually at their handler’s residence or in a group kennel. Residential kennels are indoor-outdoor metal kennels raised 2 feet from the ground, giving the dog the option of sleeping inside or outside. Group kennels are indoor-outdoor, concrete kennels, and dogs are confined inside during the night.” “…blood samples from DHS working dogs during November 2015 or April 2016” “Triatomine bugs were opportunistically collected by dog handlers in summer 2016 from group kennels, outside handler’s residence around canine housing, and at stations where dogs worked.” |
| Participants | 6 | (*a*) Give the eligibility criteria, and the sources and methods of selection of participants | 7-8 | “Working dogs were sampled from all 5 management areas, with a goal of sampling at least 60% of the dogs that occurred within each management area. Additionally, we sampled DHS canines that were in training or waiting for deployment at a training facility in management area #1 (Fig. 1). Sample criteria included dogs over 6 months in age and on active duty or in training.” |
| Variables | 7 | Clearly define all outcomes, exposures, predictors, potential confounders, and effect modifiers. Give diagnostic criteria, if applicable | 8-10, 12 | “Serum and plasma samples were screened for anti-*T. cruzi* antibodies by Chagas Stat-Pak® rapid immunochromatographic test (ChemBio, NY) …samples were tested according to manufacturer’s protocol and read for result determination after 15 minutes. Bands were scored from 1-4 depending on intensity of color, with faint bands being scored as ‘1’ and bold band being scored as ‘4’. Additionally, very faint bands that were not perceptible enough to be categorized as ‘1’, yet with some low level of color development to differentiate them from negative, were tracked as ‘inconclusive’ and subjected to additional testing.” “All positive or inconclusive samples as determined by Stat-Pak® plus 10% of the negatives were tested by both indirect fluorescent antibody (IFA) test and Trypanosoma Detect™ (InBios, International, Inc., Seattle, WA)…Titer values of 20 or higher were considered positive and readers were blinded to previous results… Serum or plasma were tested according to manufacturer's protocol [Trypanosoma Detect™] and read for result determination after 20 minutes. Bands were scored using the same metric of intensity as used for the Chagas Stat-Pak®, with very faint bands tracked as ‘inconclusive’. Serological positivity was assigned to samples that tested positive on at least two independent tests.”” Samples with Ct values less than 34 were considered suspect positive and subjected to further testing.” “Samples that yielded a band of the appropriate size were interpreted as positive in our analyses. Parasite positive dogs were defined as those that tested positive on both the rt-PCR screening and PCR assays.” “Samples with Ct values less than 34 were considered positive, and fluorescence signal determined the strain type” “Due to the uncertainty of sample serostatus associated with the inconclusive band development, antibody-positive dogs were defined using two methods; a) in the conservative method, inconclusive band development was interpreted as negative, and b) in the inclusive method, inconclusive band development was interpreted as positive.” |
| Data sources/ measurement | 8* | For each variable of interest, give sources of data and details of methods of assessment (measurement). Describe comparability of assessment methods if there is more than one group | 7,8,11 | “We used a cross sectional study design to collect blood samples from DHS working dogs during November 2015 or April 2016” “Demographic information was collected on all dogs sampled including age, sex, breed, canine job, sleeping location and station of duty.” “Triatomine bugs were opportunistically collected by dog handlers in summer 2016 from group kennels, outside handler’s residence around canine housing, and at stations where dogs worked.” |
| Bias | 9 | Describe any efforts to address potential sources of bias |  |  |
| Study size | 10 | Explain how the study size was arrived at | 7-8 | “We used a cross sectional study design to collect blood samples from DHS working dogs during November 2015 or April 2016.Working dogs were sampled from all 5 management areas, with a goal of sampling at least 60% of the dogs that occurred within each management area.” |
| Quantitative variables | 11 | Explain how quantitative variables were handled in the analyses. If applicable, describe which groupings were chosen and why | 12 | Serostatus and parasitemia status were treated as POS/NEG using two methods. “Due to the uncertainty of sample serostatus associated with the inconclusive band development, antibody-positive dogs were defined using two methods; a) in the conservative method, inconclusive band development was interpreted as negative, and b) in the inclusive method, inconclusive band development was interpreted as positive. In the absence of gold standard serological methodology, these two different criteria of positivity (method A and B) were analyzed separately to provide a range of results.”  Canine age was binned as follows: young=6 months to <3 years, middle age=> 3 years to <6 years, senior=> 6 years. Data was analysed with and without binning and no difference in age was found. |
| Statistical methods | 12 | (*a*) Describe all statistical methods, including those used to control for confounding | 12-13 | “Bivariable analysis using the chi-squared or Fisher’s exact was used to identify putative risk factors. Factors with a p< 0.25 from the initial screening were used in a logistic regression model, while controlling for management area as a random effect. Generalized linear mixed models were calculated and factors with values of p < 0.05 were considered significant. Odds ratios and 95% confidence intervals were calculated. To determine variation in seroprevalence across management areas, a logistic regression model was used in which the training center served as the referent to which all five management areas were compared.” |
|  |  | (*b*) Describe any methods used to examine subgroups and interactions |  | “Factors with a p< 0.25 from the initial screening were used in a logistic regression model, while controlling for management area as a random effect.” |
|  |  | (*c*) Explain how missing data were addressed |  | N/A |
|  |  | (*d*) If applicable, describe analytical methods taking account of sampling strategy |  | N/A |
|  |  | (*e*) Describe any sensitivity analyses |  | N/A |
| Results | | |  |  |
| Participants | 13* | (a) Report numbers of individuals at each stage of study—eg numbers potentially eligible, examined for eligibility, confirmed eligible, included in the study, completing follow-up, and analysed | 17-19 | “In comparing the results across all three serological testing platforms (Table 4), all IFA positive samples are positive on Trypanosoma Detect™, and all but two samples are Stat-Pak® positive-both of these samples having a titer of 20. When comparing the IFA negative samples 71.3% are positive or inconclusive on Stat-Pak® and 48.4% are positive or inconclusive on Trypanosoma Detect™. The majority of the IFA-positive dogs were strongly positive (band score of 3 or 4) on Stat- Pak (68%) and Trypanosoma Detect™ (78.9%). Of the 57 randomly-selected Stat-Pak® negative samples that were subjected to additional serologic testing, one was positive on both IFA (titer 20) and Trypanosoma Detect™; this sample was counted as positive in the seroprevalence estimates.”  “The first PCR-positive dog was sampled from area # 5 in November and was positive for antibodies by all three serology assays with a relatively high titer (640) on IFA. Using the multiplex real time PCR to determine *T. cruzi* DTUs, we found that this dog harbored DTU TcIV. The second PCR-positive dog was from the canine training center, sampled in April, positive on all serology assays with a titer of 320 and harbored a mix TcI/TcIV. The third dog was from area # 2, sampled in April, was negative by all serological assays, and strain type could not be determined.” |
|  |  | (b) Give reasons for non-participation at each stage | 18, 19 | “Nine (15.8%) samples that were both Stat-Pak® and IFA negative were positive on Trypanosoma Detect™; these dogs were counted as negative in the seroprevalence estimates, but could be false negatives.”  “The third dog… was negative by all serological assays, and strain type could not be determined. When this PCR positive yet serologically-negative dog was included in binomial analysis of risk factors and the logistical regression model, no difference was found in in significant associations.” 1 PCR positive, seronegative dog was not included in analysis of seropositive dogs. |
|  |  | (c) Consider use of a flow diagram | NA | NA |
| Descriptive data | 14* | (a) Give characteristics of study participants (eg demographic, clinical, social) and information on exposures and potential confounders | 13 | “Distribution of samples among the five management areas ranged from 47 (8.9%) to 135 (25.6%), and 86 (16.3%) dogs were sampled from the training center. The most common breeds were Belgian Malinois and German Shepherd, which together comprised 86% of the sampled dogs, with Dutch Shepherds, Sable Shepherds, Groenendael and Labrador Retriever comprising the remainder. Age ranged from approximately 6 months to 13 years with a median of 4.47 and a mean of 4.79. There were 351 males (66.5%) and 177 females (33.5%). Of the dogs sampled, 55.9% spend their off-duty time in individual residential kennels whereas 44.1% were group kenneled. The sample sizes of dogs within each canine job category are not disclosed because it is law enforcement sensitive information. ” |
|  |  | (b) Indicate number of participants with missing data for each variable of interest | NA | NA |
| Outcome data | 15* | Report numbers of outcome events or summary measures | 14, 16 | “In considering inconclusive bands on immunochromatographic tests as negative, 39 of 528 (7.4%) of dogs were seropositive for antibodies to T. cruzi on at least 2 assays. Across management areas and the training center, seroprevalence ranged from 4.3% to 10% (Figure 1).” “In considering inconclusive bands on serologic tests as positive, 100 of 528 (18.9%) of dogs were seropositive for antibodies to *T. cruzi* on at least 2 assays. Seroprevalence ranged from 11.6% to 26.7% across management areas and the training center (Figure 1).” “*T. cruzi* DNA was detected in the buffy coat fraction of the blood in three of 528 (0.6%) dog samples according to our diagnostic method which included amplification in both a screening and confirmatory assay. “Nine (45%) triatomines were positive for *T. cruzi* including half of the *T. gerstaeckeri* specimens but neither of the two *T. rubida* specimens” |
| Main results | 16 | (*a*) Give unadjusted estimates and, if applicable, confounder-adjusted estimates and their precision (eg, 95% confidence interval). Make clear which confounders were adjusted for and why they were included | 15, 16 | “Multivariable logistic regression analysis showed a significant association (odds ratio [OR] 0.41, 95% CI 0.17-0.99, p=0.047, Table 2) between breed and seropositive dogs, after controlling for management areas as a random effect (Table 2).” “…seroprevalence did not significantly differ across management areas and the training center when positivity was defined according to Method A, dogs from management area #2 (OR 2.6, 95% CI 1.0-6.7, p=0.04) and #3 (OR 2.8, 95% CI 1.2-6.8, p=0.02) had significantly higher seroprevalence compared to the training center when seropositivity was determined according to Method B (Table 3)..” |
|  |  | (*b*) Report category boundaries when continuous variables were categorized | 12 | “Assessed variables were dog age (young=6 months to <3 years, middle age=> 3 years to <6 years, senior=> 6 years)...” |
|  |  | (*c*) If relevant, consider translating estimates of relative risk into absolute risk for a meaningful time period |  | NA |
| Other analyses | 17 | Report other analyses done—eg analyses of subgroups and interactions, and sensitivity analyses |  | NA |
| Discussion | | |  |  |
| Key results | 18 | Summarise key results with reference to study objectives | 21, 24, 25 | “Exposed dogs were present in all five management areas and the canine training school, with an overall apparent seroprevalence of 7.4-18.9%.” “We found three dogs (0.6%) harbored parasite PCR in their blood, suggesting that these dogs are parasitemic…two dogs with successfully typed infections harbored DTUs TcIV and a TcI/TcIV mix, consistent with previous studies on dogs in the US [55,56].” “We found an infection prevalence of 45% in the kissing bugs collected from areas where the working dogs frequent, including kennels, stations and handler’s residence, including DTUs TcI, TcIV, and TcI/IV mix.” |
| Limitations | 19 | Discuss limitations of the study, taking into account sources of potential bias or imprecision. Discuss both direction and magnitude of any potential bias | 23 | “…some previous canine studies have counted faint bands as negative [6,9] while others have interpreted them as positive for analysis [43]. Our presentation of a seroprevalence calculated both conservatively (very faint bands interpreted as negative) and inclusively (very faint bands interpreted as positive) is an effort to account for imperfect diagnostics. Until refined T. cruzi diagnostic tools are available, we encourage transparency in presenting results on single vs. multiple tests across all strengths of test response.” |
| Interpretation | 20 | Give a cautious overall interpretation of results considering objectives, limitations, multiplicity of analyses, results from similar studies, and other relevant evidence | 22 | In comparison to the other cited studies, “These studies and ours suggest that despite the regular veterinary care, quality food and shelter, highly-valued working dogs can have similar or greater *T. cruzi* infection than stray and shelter dogs in the US and free roaming or pet dogs in endemic countries.” |
| Generalisability | 21 | Discuss the generalisability (external validity) of the study results | 22-23 | “A key contrast in the role of canines in the *T. cruzi* transmission cycle in central Texas versus the role in areas throughout Latin America is tied to the differences in the human-dog relationship as well as socioeconomic/housing differences and bug ecology. Although dogs have been shown to be important *T. cruzi* reservoir hosts in areas of Latin America [58,64,65], dogs in central Texas are typically housed either in a kennel separate and somewhat distant from the human dwelling or indoors in a house constructed with screens and doors that limit bug entry.” |
| Other information | | |  |  |
| Funding | 22 | Give the source of funding and the role of the funders for the present study and, if applicable, for the original study on which the present article is based | 27 | “This material is based upon work supported by the U.S. Department of Homeland Security task order HSHQDC-15-J-00217. The views expressed in this article are those of the authors and do not necessarily represent the views of the Department of Homeland Security or the United States Government. The funders had no role in study design, data collection and analysis, decision to publish, or preparation of the manuscript.” |

*Give information separately for exposed and unexposed groups.

**Pagination is based on the double-spaced, complete manuscript, as submitted for consideration for publication.

**Note:** An Explanation and Elaboration article discusses each checklist item and gives methodological background and published examples of transparent reporting. The STROBE checklist is best used in conjunction with this article (freely available on the Web sites of PLoS Medicine at http://www.plosmedicine.org/, Annals of Internal Medicine at http://www.annals.org/, and Epidemiology at http://www.epidem.com/). Information on the STROBE Initiative is available at www.strobe-statement.org.
